# Multimodal artificial intelligence assessment of simulated cardiac arrest performance: a pilot study

**DOI:** 10.1016/j.resplu.2026.101517

**Published:** 2026-10-06

**Authors:** Pierre-Marc Dion, Jennifer Cardenas-Mancera, Claire Touchie, Vicki LeBlanc, Arnaud Mbadjeu, Curtis Nickel, Richard Waldolf, YeonJung Yoo, Lucas Bergamascki, Pooyan Sekhavati, Simone Crooks, Yuqi Gu

**Affiliations:** a Acute Care Research Program, Ottawa Hospital Research Institute, Ottawa, Ontario, Canada; b Department of Family Medicine, McGill University, Montreal, Quebec, Canada; c Department of Anesthesiology and Pain Medicine, University of Ottawa, Ottawa, Ontario, Canada; d Department of Medicine, University of Ottawa, Ottawa, Ontario, Canada; e Centre for Innovation in Medical Education, University of Ottawa, Ottawa, Ontario, Canada; f Department of Innovation in Medical Education, University of Ottawa, Ottawa, Ontario, Canada

**Keywords:** Artificial intelligence, Cardiac arrest, Cardiopulmonary resuscitation, Simulation, Medical education, Assessment

## Abstract

**Objectives:**

Assessment of simulated cardiac arrest performance is resource-intensive and subject to inter-rater variability. We evaluated whether a multimodal artificial intelligence (AI) system could score video-recorded cardiac arrest megacode simulations with agreement comparable to expert raters.

**Methods:**

Six scripted megacode simulations (three ventricular fibrillation, three pulseless electrical activity) representing exemplar, guideline-deviation and non-technical-deficiency performance were scored by two ACLS instructors using a validated team-leader checklist; consensus ratings served as the reference. Two operators analysed each video with Gemini 1.5 Pro at three temperature settings (504 item-level ratings). We calculated Cohen’s kappa, Gwet’s AC1, and sensitivity and specificity for identifying performed and omitted actions, and compared AI self-reported confidence between correct and incorrect ratings.

**Results:**

Expert inter-rater agreement was *κ* = 0.78 (95% CI 0.64–0.93). AI–expert agreement was *κ* = 0.62 (95% CI 0.49–0.75; range 0.40–0.86 across configurations) and AC1 = 0.77. The AI identified performed actions with high sensitivity (0.98, 95% CI 0.96–0.99) but detected omitted actions poorly (specificity 0.58, 95% CI 0.44–0.72), particularly omissions defined by timing or sequence. Temperature had no consistent effect. Incorrect ratings of actions as performed carried lower mean self-reported confidence than correct ones (93.6 vs 98.1), but 48% were made with maximal confidence.

**Conclusions:**

A multimodal AI system reliably confirmed completed actions but frequently failed to detect omitted actions, yielding moderate overall agreement that was below expert–expert agreement. Current systems may support, but not replace, expert assessment of cardiac arrest simulation.

## Highlights

• Multimodal AI scored video-recorded cardiac arrest megacode simulations.
• AI detected performed actions (sensitivity 0.98) but missed many omissions (specificity 0.58).
• AI–expert agreement (*κ* 0.62) was below expert–expert agreement (*κ* 0.78).
• Overcalls had lower AI confidence, but half were made with maximal confidence.

## Introduction

Simulation-based education enables healthcare professionals to practice technical and non-technical skills in controlled environments, supporting deliberate practice and performance assessment.^1^, ^2^ Advanced cardiac life support (ACLS) training uses simulation to reinforce guideline-based management and team coordination during cardiac arrest and has been associated with improved resuscitation performance and communication.^2^ Despite these benefits, assessment of simulation performance commonly relies on expert raters and may be resource-intensive and subject to inter-rater variability.^3^, ^4^

Structured checklists and validated assessment tools have improved consistency in simulation-based assessment; however, challenges related to scalability and reproducibility remain.^4^ These limitations are particularly relevant in emergency medicine training, where repeated assessment of resuscitation performance is required and faculty resources may be constrained.^5^

AI has been evaluated as a scorer in objective structured clinical examinations (OSCEs), with mixed agreement with human examiners.^6^, ^7^ When multimodal models scored video-recorded OSCE skills, including basic life support, they generally assigned higher scores than human examiners and agreed less with them on steps requiring auditory cues.^8^ Vision-language and computer-vision models have analysed surgical and trauma resuscitation video,^9^, ^10^ and language models have evaluated text-based resuscitation scenarios,^11^, ^12^, ^13^ but video-based AI assessment of cardiac arrest simulation remains unevaluated.

This pilot study evaluated whether a multimodal AI system could assess cardiac arrest simulation performance with agreement comparable to expert raters, and characterised its errors, including its ability to detect omitted actions and the value of its self-reported confidence.

## Materials and methods

### Study design

This experimental pilot study evaluated agreement between a multimodal AI system and expert consensus ratings in scoring prerecorded cardiac arrest megacode simulations. The study was approved by the Ottawa Health Science Network Research Ethics Board (March 5, 2025; #20250047-01H) and reported in accordance with the Reporting Guidelines for Health Care Simulation Research.^14^ AI operators were blinded to expert reference scores.

### Scenarios and recording

Six scenarios were written for this study: three ventricular fibrillation (VFib) and three pulseless electrical activity (PEA), representing exemplar performance, technical (guideline) deficiencies and non-technical deficiencies. Each script specified a patient stem, timed deviations and a predetermined checklist answer key (Table S1). Faculty and residents performed the scenarios on April 24, 2025 at the University of Ottawa Skills and Simulation Centre using a high-fidelity SimMan 3G manikin (Laerdal Medical Canada Ltd, Toronto, ON, Canada). Scenarios (approximately 10 min) were recorded without editing using a fixed-position wide-angle camera (DJI Action 2, DJI, Shenzhen, China) with built-in microphones capturing audio (Fig. S1). Only team-leader actions were assessed, as in the megacode checklist used for ACLS certification.^4^

### Reference standard

Two ACLS instructors who routinely use the megacode rubric independently scored each recording from video using a binary (yes/no) checklist of 16 VFib or 12 PEA items (Table S2).^4^ They were aware of the study aims but blinded to the scripts and answer keys, and received no additional calibration. Disagreements were resolved through discussion, and the consensus ratings served as the reference standard.

### Intervention

Recordings were analysed in May 2025 with Gemini 1.5 Pro (Google DeepMind; Google AI Studio), the only publicly available model we identified that accepted video with audio as direct input. A five-step prompt (Table S3) provided the AHA ACLS guidelines, the patient stem, the video and the checklist, then requested a yes/no rating, reasoning and self-reported confidence (0–100) for each item, and an overall narrative that was not evaluated.

Temperature controls output randomness, from near-deterministic (0) to most variable (2.0). Two operators independently analysed each de-identified video at temperatures of 0, 1.0 and 2.0 with other settings at default (36 runs; 504 item-level ratings), each run in a new study-specific account and session with activity logging disabled (Table S3). Each video corresponded to approximately 177,000 input tokens at published rates.

### Outcomes and analysis

The primary outcome was agreement between AI and expert consensus ratings, measured with Cohen's kappa, interpreted using McHugh's thresholds (0.40–0.59 weak, 0.60–0.79 moderate, ≥0.80 strong),^15^ and Gwet's AC1, which is less affected by prevalence.^16^ Treating “performed” as positive, we calculated sensitivity, specificity, positive and negative predictive values (PPV, NPV) and a constant “always performed” benchmark, with and without exemplar scenarios, which contained no omitted actions. Ratings were pooled across operators and temperatures, with 95% confidence intervals (CIs) from a cluster bootstrap over the 84 scenario-items. We also compared AI agreement with each expert against expert–expert agreement.

For each item, the average AI score was the proportion of the six runs (two operators × three temperatures) rating the action as performed, reported alongside the expert consensus (Table 1). Specificity was compared descriptively across temperature settings. Mean self-reported confidence was compared between correct and incorrect ratings, and between matched and mismatched ratings by arrest type using independent-samples t-tests (Tables S5 and S6). Analyses used Microsoft Excel and Python 3.11 (scikit-learn, SciPy); *p* < 0.05 was significant. No formal sample size calculation was performed given the pilot nature of this study.

**Table 1:** Average AI score and expert consensus score for each checklist item.

	**Average AI score (proportion of 6 runs rated performed)**			**Expert consensus (1 = performed, 0 = omitted)**		
**A. Pulseless electrical activity (PEA) checklist item**	**Exemplar**	**Guideline**	**Non-technical**	**Exemplar**	**Guideline**	**Non-technical**
Team leader assigns team member roles	1.00	1.00	0.00	1	1	0
Team leader always ensures high-quality CPR	1.00	0.50	0.83	1	0	0
Team leader ensures team members communicate well	1.00	1.00	0.50	1	1	0
Team leader recognizes pulseless electrical activity	1.00	1.00	1.00	1	1	1
Team leader verbalizes potential reversible causes in the form of H’s and T’s	1.00	1.00	0.33	1	1	0
Team leader ensures that CPR is immediately resumed after the rhythm check	1.00	0.83	1.00	1	0	1
Team leader ensures there is appropriate airway management	1.00	0.83	1.00	1	0	1
Team leader administers appropriate drug (s)	1.00	1.00	1.00	1	1	1
Team leader administers appropriate dose of drugs	1.00	0.00	1.00	1	0	1
Team leader identifies return of spontaneous circulation	1.00	1.00	1.00	1	1	1
Ensures BP and 12-lead ECG are performed, O_2_ saturation is monitored, verbalizes need for endotracheal intubation and waveform capnography, and orders laboratory tests	0.83	0.50	1.00	1	0	1
Considers targeted temperature management	1.00		0.83	1	0	1

**B. Ventricular fibrillation (VFib) checklist item**	**Exemplar**	**Guideline**	**Non-technical**	**Exemplar**	**Guideline**	**Non-technical**

Team leader assigns team member roles	1.00	1.00	0.00	1	1	0
Team leader always ensures high-quality CPR	1.00	0.00	0.00	1	0	0
Team leader ensures team members communicate well	1.00	0.67	0.17	1	1	0
Team leader recognizes pulseless ventricular fibrillation	1.00	1.00	1.00	1	0	1
Team leader immediately defibrillates patient	1.00	0.67	1.00	1	0	1
Team leader uses correct energy setting used for defibrillation	1.00	0.17	1.00	1	0	1
Team leader verbalizes potential reversible causes in the form of H’s and T’s	1.00	1.00	1.00	1	1	1
Team leader ensured everyone was clear before shock	1.00	0.67	0.50	1	0	0
Team leader ensures that CPR is resumed immediately after shocks	1.00	0.83	0.83	1	0	1
Team leader ensures there is appropriate airway management	1.00	1.00	1.00	1	1	1
Team leader ensures that appropriate cycles of drugs-rhythm check/shock – CPR	1.00	0.83	0.17	1	0	0
Team leader administers appropriate drug (s)	1.00	0.50	0.83	1	0	1
Team leader administers appropriate drug doses	1.00	0.00	1.00	1	0	1
Team leader identifies return of spontaneous circulation	1.00	1.00	1.00	1	0	1
Ensures BP and 12-lead ECG are performed, O_2_ saturation is monitored, verbalizes need for endotracheal intubation and waveform capnography, and orders laboratory tests	1.00	1.00	0.83	1	1	1
Considers targeted temperature management	1.00	0.17	1.00	1	0	1

Average AI score = proportion of six runs (two operators × three temperatures) in which the AI rated the action as performed (1 = performed in all runs, 0 = in none). Agreement is perfect when the AI score equals the expert score (1.00 with 1, or 0.00 with 0); the greater the difference, the lower the agreement. Exemplar = exemplar performance; Guideline = technical (guideline) deficiencies; Non-technical = non-technical deficiencies. CPR = cardiopulmonary resuscitation; ROSC = return of spontaneous circulation.

## Results

Inter-rater agreement between experts was *κ* = 0.78 (95% CI 0.64–0.93; 8 of 84 items discordant), and consensus matched the predetermined answer key on 77 of 84 items (*κ* = 0.81).

Pooled AI–expert agreement was moderate (*κ* = 0.62, 95% CI 0.49–0.75; AC1 0.77, 0.64–0.87), ranging from 0.40 to 0.86 across configurations (Table S4). AI agreement with each expert (*κ* = 0.53 and 0.59) was below expert–expert agreement.

The AI identified performed actions with a sensitivity of 0.98 (95% CI 0.96–0.99) but omitted actions with a specificity of 0.58 (0.44–0.72); PPV was 0.84 and NPV 0.93 (Table 2). Accuracy was 0.86 versus 0.69 for a constant “always performed” classifier (AC1 0.77 vs 0.58). Excluding exemplar scenarios, *κ* was 0.56 and AC1 0.60 (constant classifier 0.28). Agreement was lowest in guideline-deviation scenarios (*κ* = 0.42; specificity 0.50).

**Table 2:** Agreement and diagnostic accuracy of AI ratings against expert consensus.

**Subset**	***N***	**Sensitivity**	**Specificity**	**PPV**	**NPV**	**Cohen *κ***
All scenarios	504	0.98 (0.96–0.99)	0.58 (0.44–0.72)	0.84 (0.76–0.91)	0.93 (0.85–0.98)	0.62 (0.49–0.75)
Excluding exemplar	336	0.97 (0.93–0.99)	0.58 (0.44–0.71)	0.72 (0.59–0.84)	0.94 (0.86–0.99)	0.56 (0.41–0.70)
Guideline deviation	168	0.97 (0.90–1.00)	0.50 (0.33–0.67)	0.56 (0.34–0.74)	0.96 (0.86–1.00)	0.42 (0.22–0.62)
Non-technical deficiency	168	0.96 (0.93–0.99)	0.72 (0.52–0.90)	0.88 (0.75–0.97)	0.91 (0.75–0.98)	0.73 (0.53–0.87)
Ventricular fibrillation	288	0.97 (0.94–1.00)	0.58 (0.40–0.76)	0.82 (0.71–0.92)	0.92 (0.79–0.99)	0.61 (0.43–0.78)
Pulseless electrical activity	216	0.99 (0.97–1.00)	0.57 (0.36–0.78)	0.86 (0.74–0.95)	0.94 (0.82–1.00)	0.63 (0.43–0.82)
Expert 1 vs Expert 2	84	–	–	–	–	0.78 (0.64–0.93)

Values are point estimates (95% CI). “Performed” was treated as positive: sensitivity = proportion of actions rated performed by experts that the AI rated performed; specificity = proportion of actions rated omitted by experts that the AI rated omitted; PPV and NPV depend on the proportion of performed actions (69% overall) and should not be generalised. Ratings pooled across two operators and three temperatures; CIs from cluster bootstrap over 84 scenario-items (5000 resamples). Gwet's AC1: overall 0.77 (0.64–0.87), excluding exemplar 0.60 (0.42–0.76); constant “always performed” classifier 0.58 and 0.28. Exemplar scenarios contained no omitted actions (sensitivity 0.99). Expert *κ* CI is asymptotic.

At the item level (Table 1), explicit or verbal omissions were consistently detected, including incorrect epinephrine dose and absent role assignment (6 of 6 runs in each scenario). Omissions involving timing or sequence were often missed. In the VFib guideline-deviation scenario, the AI rated rhythm recognition and return of spontaneous circulation (ROSC) identification as performed in all six runs, although the experts rated both as omitted; in every other scenario, the AI rated these items correctly. In the same scenario, delayed defibrillation, delayed resumption of CPR and incorrect drug-rhythm cycles were detected in only one or two of the six runs. The model's written reasoning sometimes noted delays yet still credited the action, and sometimes described unobserved actions with timestamps (Table S7).

Specificity did not change consistently with temperature (0.54, 0.58 and 0.62 at 0, 1.0 and 2.0). Among 407 “performed” ratings, overcalls had lower mean confidence than correct ratings (93.6 vs 98.1), but 32 of 66 overcalls (48%) had confidence of 100. Confidence was significantly lower for mismatched than matched ratings in four of six PEA configurations and none of six VFib configurations (Tables S5 and S6).

## Discussion

The AI reliably confirmed performed actions but detected only about six in ten omissions. Agreement was moderate overall, below expert–expert agreement, and weak in guideline-deviation scenarios. Overall agreement was inflated by the exemplar scenarios and by the high proportion of performed actions, highlighting the value of reporting diagnostic and prevalence-robust metrics. These observations suggest two possible mechanisms for overcalling: a more literal reading of the checklist than the experts', and confident descriptions of expected actions that were not observed. The latter is consistent with evidence that video-language models describe events suggested by their prior knowledge rather than the video^17^ and that localising actions in time remains challenging.^18^ Multimodal models scoring video-recorded OSCEs similarly over-scored.^8^ Checklists with explicit timing criteria and prompts requiring time-stamped evidence may reduce these errors.

Self-reported confidence offered only a modest signal: with half of overcalls made at maximal confidence, thresholds could prioritise items for human review but are not a sufficient safeguard. The absence of a consistent temperature effect agrees with previous work.^19^

### Strengths and limitations

Strengths include scripted scenarios with predetermined answer keys verified by blinded experts, two independent operators, three temperature settings and a validated rubric. Limitations include six scenarios performed by faculty and residents at one centre, limiting generalisability. A single camera and microphone may have missed information, particularly verbal communication, and only team-leader actions were assessed. The raters knew the study aims, were not formally calibrated and disagreed on eight items, limiting the precision of the reference standard. A single model and a single prompt were evaluated; its version was not recorded and it is no longer available. Finally, we evaluated post hoc, operator-mediated assessment only, not real-time operation, team assessment, feedback quality or educational impact.

### Implications

AI may eventually support scalable assessment, for example by drafting checklist ratings for expert review, but current detection of omitted actions does not support unsupervised use. Simulation-based education also depends on formative feedback, and whether AI can generate accurate, educationally useful feedback requires separate evaluation. Future work should use larger datasets, compare models, and test rubrics and prompts designed to capture timing.

## Conclusions

A multimodal AI system reliably confirmed completed actions in cardiac arrest simulation but frequently missed omitted actions, with moderate agreement below expert–expert agreement. These findings identify specific targets for refinement before AI-assisted assessment is implemented.

## Declaration of generative AI and AI-assisted technologies in the manuscript preparation process

During the preparation of this manuscript the authors used Claude, Anthropic to assist with statistical re-analysis, grammatical edits and table preparation. The authors reviewed and edited the content as needed and take full responsibility for the content of the publication

## CRediT authorship contribution statement

**Pierre-Marc Dion:** Conceptualization, Formal analysis, Investigation, Methodology, Writing – original draft. **Jennifer Cardenas-Mancera:** Conceptualization, Funding acquisition, Investigation, Methodology, Writing – original draft, Writing – review & editing. **Claire Touchie:** Conceptualization, Funding acquisition, Methodology, Supervision, Writing – review & editing. **Vicki LeBlanc:** Conceptualization, Data curation, Formal analysis, Funding acquisition, Investigation, Methodology, Writing – review & editing. **Arnaud Mbadjeu:** Conceptualization, Funding acquisition, Methodology, Software, Writing – review & editing. **Curtis Nickel:** Conceptualization, Funding acquisition, Methodology, Writing – review & editing. **Richard Waldolf:** Funding acquisition, Investigation, Methodology, Validation. **YeonJung Yoo:** Data curation, Formal analysis, Methodology, Project administration, Software, Writing – review & editing. **Lucas Bergamascki:** Project administration, Software, Writing – review & editing. **Pooyan Sekhavati:** Data curation, Methodology, Project administration, Software, Writing – review & editing. **Simone Crooks:** Funding acquisition, Methodology, Writing – review & editing. **Yuqi Gu:** Conceptualization, Data curation, Formal analysis, Funding acquisition, Investigation, Methodology, Project administration, Writing – review & editing.

## Funding

This study was funded by the Centre for Innovation in Medical Education (CIMED), University of Ottawa. Yuqi Gu, Arnaud Mbadjeu, Curtis Nickel, and Simone Crooks received institutional support from the Ottawa Hospital Anesthesia Alternate Funds Association. The funding sources had no role in the study design; data collection, analysis, or interpretation; preparation of the manuscript; or the decision to submit the article for publication.

## Declaration of competing interest

The authors declare that they have no known competing financial interests or personal relationships that could have appeared to influence the work reported in this article.

## Appendix A Supplementary material

Supplementary Data 1 Figure S1 (recording setup) and Tables S1–S7: scenario scripts and answer keys, megacode checklist items, AI prompt and settings, agreement by configuration, AI confidence analyses for pulseless electrical activity and ventricular fibrillation, and illustrative examples of AI reasoning.
