# Supplementary material for: Multimodal artificial intelligence assessment of simulated cardiac arrest performance: a pilot study
Source: Resusc Plus. 2026 Oct 6;32:101517. doi: 10.1016/j.resplu.2026.101517 (PMC13697277; doi:10.1016/j.resplu.2026.101517)
Supplement: Supplementary file 1 [file mmc1.docx]

**Supplemental digital content**

Figure S1: Camera position and field of view.

Table S1: Cardiac arrest scenarios and scripted performance.

Table S2: Mega-code assessment rubrics.

Table S3: Prompting sequence.

Table S4: Agreement between AI and expert ratings by arrest type, AI operator and temperature.

Table S5: Artificial intelligence confidence scores for pulseless electrical activity scenarios.

Table S6: Artificial intelligence confidence scores for ventricular fibrillation scenarios.

Table S7: Illustrative examples of AI reasoning.

**Figure S1.** Camera position and field of view. De-identified still image from one recording, showing the fixed wide-angle camera (DJI Action 2) view of the team leader, team and manikin used for all scenarios.

**Table S1.** Cardiac arrest scenarios and scripted performance. Scenarios were performed by faculty and residents following written event timelines; expert raters were blinded to this information.

| **Scenario** | **Scripted performance and deviations** |
| --- | --- |
| Ventricular fibrillation — exemplar | Standard ACLS performance: roles assigned at the start; defibrillator attached and first shock (200 J) within about 1 minute of VF recognition; CPR resumed within 5 seconds of each shock; rhythm checks every 2 minutes; epinephrine 1 mg IV every 3–5 minutes; airway progressed from BVM to supraglottic device; "Clear!" before each shock; H's and T's verbalized; CPR quality and fatigue monitored; ROSC recognized and post-arrest care initiated. |
| Ventricular fibrillation — technical deficiencies | At the first rhythm check the defibrillator was not charged and the pads were misplaced; first shock delayed and delivered at incorrect energy (100 J); no "Clear!" before the shock; CPR resumed only after a delay of about 20 seconds; CPR rate about 50/min; epinephrine given at a 10-fold under-dose (0.1 mg IV); rhythm checks every 3 minutes; no advanced airway after the second cycle; amiodarone not given, team indecisive. |
| Ventricular fibrillation — non-technical deficiencies | Technical care adequate but no roles assigned and no clear team leader (overlapping tasks, e.g., two people preparing medications); "Clear!" not consistently verbalized; CPR interruptions over 10 seconds during transitions; no discussion of H's and T's; no reassessment or coaching of CPR quality; compressor rotation not planned; decisions made by multiple voices without coordination. |
| Pulseless electrical activity — exemplar | PEA correctly identified with no shock delivered; immediate high-quality CPR with feedback; epinephrine 1 mg IV early and repeated; rhythm and pulse checks every 2 minutes; H's and T's reviewed, with hypovolemia suspected and IV fluids given; airway advanced from OPA to supraglottic device; clear role delegation and closed-loop communication. |
| Pulseless electrical activity — technical deficiencies | CPR start delayed more than 30 seconds after pulselessness; defibrillator charged and a shock delivered for PEA; CPR restarted at about 80/min with shallow depth and frequent pauses; epinephrine given at a 10-fold under-dose (0.1 mg); rhythm check performed without a pulse check; no airway secured until after 6 minutes; no IV fluids despite suspected hypovolemia. |
| Pulseless electrical activity — non-technical deficiencies | No role assignment and no designated leader, with conflicting commands; epinephrine given without announcement, with confusion about whether it was administered; rhythm check delayed while the team argued about next steps; fatigued compressor not rotated; H's and T's never discussed; no closed-loop communication; confusion over whether a pulse had returned. |

**Table S2.** Mega-code assessment rubrics.

| **Ventricular fibrillation checklist** | **No = 0, Yes = 1** |
| --- | --- |
| Team leader assigns team member roles |  |
| Team leader always ensure high-quality CPR |  |
| Team leader ensures team members communicate well |  |
| Team leader recognizes pulseless ventricular fibrillation |  |
| Team leader immediately defibrillates patient |  |
| Team leader uses correct energy settings used for defibrillation |  |
| Team leader ensured everyone was clear before shock |  |
| Team leader ensures that CPR is resumed immediately after shocks |  |
| Team leader ensures there is appropriate airway management |  |
| Team leader ensures that appropriate cycles of drugs-rhythm check/shock - CPR |  |
| Team leader administers appropriate drugs |  |
| Team leader administers appropriate dose of drugs |  |
| Team leader verbalizes potential causes in the form of H’s and T’s |  |
| Team leader identifies return of spontaneous circulation |  |
| Ensures BP and 12-lead ECG are performed, O2 saturation is monitored, verbalizes need for endotracheal intubation and waveform capnography, and orders laboratory tests |  |
| Considers targeted temperature management |  |

| **Pulseless electrical activity checklist** | **No = 0, Yes = 1** |
| --- | --- |
| Team leader assigns team member roles |  |
| Team leader always ensures high-quality CPR |  |
| Team leader ensures team members communicate well |  |
| Team leader recognizes pulseless electrical activity |  |
| Team leader ensures that CPR is immediately resumed after the rhythm check. |  |
| Team leader ensures there is appropriate airway management |  |
| Team leader administers appropriate drugs |  |
| Team leader administers appropriate dose of drugs |  |
| Team leader verbalizes potential causes in the form of H’s and T’s |  |
| Team leader identifies return of spontaneous circulation |  |
| Ensures BP and 12-lead ECG are performed, O2 saturation is monitored, verbalizes need for endotracheal intubation and waveform capnography, and orders laboratory tests |  |
| Considers targeted temperature management |  |

**Table S3.** Prompting sequence.

| **Step** | **Prompt content (verbatim)** | **Rationale** |
| --- | --- | --- |
| Contextual setup | "You are an ACLS simulation instructor who will be assessing participants in a standardized simulated ACLS scenario. I will be uploading the most up to date ACLS guidelines and simulation scenario stem."  ACLS Guidelines: [current AHA ACLS guidelines]  Simulation scenario: [patient scenario stem] | Establishes the framework and guidelines the AI should apply. |
| Reference scenario | "I will be uploading a simulated video of a doctor undergoing the simulation scenario provided in the reference documents. Please refrain from any analysis at this stage." [video uploaded] | Provides the video to be analysed. |
| Assessment rubric | "I will be uploading an ACLS assessment checklist." [megacode checklist, Table S2; No = 0, Yes = 1] | Directs the AI to the observable actions to be assessed. |
| Item-level assessment | "Using the assessment checklist, please assess the performance of the simulated video. For each item, explain the reasoning behind your decision. Can you also provide a confidence score (0–100%) for each checklist item. Please provide in a table format." | Binary rating of each checklist item, with justification and self-reported confidence. |
| Overall assessment | "Please provide an overall assessment of this doctor, highlighting strength and areas for improvement." | Narrative summary; not evaluated in this study. |

Input for each run: the de-identified video recording (about 10 minutes, including the audio track from the camera's built-in microphones) and the prompts above, given in sequence. The scripted deviations, event timeline and answer key were not provided. Model: Gemini 1.5 Pro via Google AI Studio, accessed May 2025; temperature 0, 1.0 or 2.0, all other settings default. Each video was assessed in a separate session, under a new study-specific Google account, with activity logging disabled and the cache cleared between assessments.

**Table S4.** Agreement between AI and expert ratings by arrest type, AI operator and temperature.

| **Arrest** | **Operator** | **Temp** | **Cohen κ vs consensus** | **P** | **Gwet AC1 vs consensus** | **κ vs Expert 1** | **κ vs Expert 2** | **κ excl. exemplar** |
| --- | --- | --- | --- | --- | --- | --- | --- | --- |
| PEA | R1 | 0 | 0.59 | <.001 | 0.79 | 0.49 | 0.59 | 0.54 |
| PEA | R1 | 1 | 0.59 | <.001 | 0.79 | 0.49 | 0.59 | 0.54 |
| PEA | R1 | 2 | 0.49 | <.001 | 0.76 | 0.40 | 0.49 | 0.44 |
| PEA | R2 | 0 | 0.68 | <.001 | 0.83 | 0.57 | 0.68 | 0.64 |
| PEA | R2 | 1 | 0.86 | <.001 | 0.91 | 0.74 | 0.86 | 0.91 |
| PEA | R2 | 2 | 0.53 | <.001 | 0.75 | 0.43 | 0.53 | 0.45 |
| VFib | R1 | 0 | 0.53 | <.001 | 0.70 | 0.39 | 0.46 | 0.44 |
| VFib | R1 | 1 | 0.40 | .001 | 0.64 | 0.37 | 0.43 | 0.31 |
| VFib | R1 | 2 | 0.75 | <.001 | 0.82 | 0.71 | 0.70 | 0.69 |
| VFib | R2 | 0 | 0.63 | <.001 | 0.76 | 0.59 | 0.56 | 0.56 |
| VFib | R2 | 1 | 0.64 | <.001 | 0.76 | 0.51 | 0.58 | 0.56 |
| VFib | R2 | 2 | 0.70 | <.001 | 0.79 | 0.56 | 0.64 | 0.62 |

R1 = AI operator 1, R2 = AI operator 2; Temp = temperature setting. PEA = pulseless electrical activity (36 ratings per row); VFib = ventricular fibrillation (48 ratings per row). P values test κ against zero. Expert 1 vs Expert 2: κ = 0.78 (95% CI 0.64–0.93), AC1 = 0.83 (84 items). κ excluding exemplar scenarios uses the guideline-deviation and non-technical scenarios only.

**Table S5.** Artificial intelligence confidence scores for pulseless electrical activity scenarios.

| **Rater** | **Mean confidence when matched with human (SD)** | **Mean confidence when differed from human (SD)** | **T statistic** |
| --- | --- | --- | --- |
| R1 – Temp 0 | 97.10 (6.93)  n = 31 | 89.00 (7.42)  n = 5 | t (34) = -2.41, P = .022 |
| R1 – Temp 1 | 97.58 (4.98)  n = 31 | 91.00 (8.94)  n = 5 | t (34) = -2.44, P = .020 |
| R1 – Temp 2 | 98.50 (2.98)  n = 30 | 88.33 (6.06)  n = 6 | t (34) = -6.31, P < .001 |
| R2 – Temp 0 | 97.34 (4.92)  n = 32 | 92.50 (5.00)  n = 4 | t (34) = -1.86, P = .072 |
| R2 – Temp 1 | 94.85 (7.54)  n = 34 | 97.50 (3.54)  n = 2 | t (34) = .49, P = .63 |
| R2 – Temp 2 | 97.00 (6.10)  n = 30 | 89.17 (11.14)  n = 6 | t (34) = -2.48, P = .018 |

R1 = AI operator 1, R2 = AI operator 2, Temp = temperature setting of the AI model during analysis. Matched/differed refers to agreement between the AI rating and the expert consensus rating for each checklist item (12 items × 3 pulseless electrical activity scenarios = 36 ratings per row). Confidence scores are on a 0–100 scale. T statistics are from independent-samples t-tests (differed minus matched).

**Table S6.** Artificial intelligence confidence scores for ventricular fibrillation scenarios.

| **Rater** | **Mean confidence when matched with human (SD)** | **Mean confidence when differed from human (SD)** | **T statistic** |
| --- | --- | --- | --- |
| R1 – Temp 0 | 96.54 (6.99)  n = 39 | 94.44 (6.82)  n = 9 | t (46) = -.81, P = .42 |
| R1 – Temp 1 | 96.76 (6.69)  n = 37 | 92.73 (6.84)  n = 11 | t (46) = -1.75, P = .088 |
| R1 – Temp 2 | 94.07 (9.59)  n = 43 | 95.00 (11.18)  n = 5 | t (46) = .20, P = .84 |
| R2 – Temp 0 | 97.56 (4.49)  n = 41 | 97.86 (3.93)  n = 7 | t (46) = .16, P = .87 |
| R2 – Temp 1 | 96.46 (5.15)  n = 41 | 95.71 (7.32)  n = 7 | t (46) = -.33, P = .74 |
| R2 – Temp 2 | 97.62 (4.02)  n = 42 | 94.17 (8.01)  n = 6 | t (46) = -1.71, P = .094 |

R1 = AI operator 1, R2 = AI operator 2, Temp = temperature setting of the AI model during analysis. Matched/differed refers to agreement between the AI rating and the expert consensus rating for each checklist item (16 items × 3 ventricular fibrillation scenarios = 48 ratings per row). Confidence scores are on a 0–100 scale. T statistics are from independent-samples t-tests (differed minus matched).

**Table S7.** Illustrative examples of AI reasoning, selected by the authors (operator 2, ventricular fibrillation technical-deficiency scenario, temperature 2; names removed). These examples were not derived from a formal qualitative analysis.

| **Checklist item** | **AI rating (confidence)** | **Expert consensus** | **AI reasoning (verbatim)** |
| --- | --- | --- | --- |
| Administers appropriate drug doses | Not performed (100) | Not performed | "No. The TL ordered incorrect doses of Epinephrine: 0.1mg (~5:19) and later 0.2mg (~7:15). The correct adult cardiac arrest dose is 1mg IV/IO every 3-5 minutes." |
| Recognizes pulseless ventricular fibrillation | Performed (90) | Not performed | "Yes. Handover stated VF. The TL eventually confirmed the rhythm visually on the monitor (~2:55) and acknowledged the patient was pulseless … Action was significantly delayed." |
| Ensures CPR is resumed immediately after shocks | Performed (100) | Not performed | "Yes. Chest compressions were promptly resumed immediately following each defibrillation attempt (~3:27, ~5:08, ~7:11)." |

The first example shows correct detection of an explicit error. The second shows a correct observation of delay but a lenient rating. The third shows a confident description that did not match the recording (the script called for a delay of about 20 seconds before CPR resumed). TL = team leader.
